# Combining data integration and molecular dynamics for target identification in α-Synuclein-aggregating neurodegenerative diseases: Structural insights on Synaptojanin-1 (Synj1)

**DOI:** 10.1016/j.csbj.2020.04.010

**Published:** 2020-04-22

**Authors:** Kirsten Jenkins, Teodora Mateeva, István Szabó, Andre Melnik, Paola Picotti, Attila Csikász-Nagy, Edina Rosta

**Affiliations:** a Randall Division of Cell and Molecular Biophysics, Institute for Mathematical and Molecular Biomedicine, King’s College London, London SE1 1UL, UK; b Department of Chemistry, King’s College London, London SE1 1DB, UK; c Institute of Biochemistry, Department of Biology, ETH Zurich, CH-8093 Zurich, Switzerland; d Faculty of Information Technology and Bionics, Pázmány Péter Catholic University, 1083 Budapest, Hungary

**Keywords:** Data integration, Molecular dynamics (MD), Neurodegenerative diseases, Parkinson’s disease (PD), Synaptojanin-1, α-Synuclein

## Abstract

Parkinson’s disease (PD), Alzheimer’s disease (AD) and Amyotrophic lateral sclerosis (ALS) are neurodegenerative diseases hallmarked by the formation of toxic protein aggregates. However, targeting these aggregates therapeutically have thus far shown no success. The treatment of AD has remained particularly problematic since no new drugs have been approved in the last 15 years. Therefore, novel therapeutic targets need to be identified and explored. Here, through the integration of genomic and proteomic data, a set of proteins with strong links to α-synuclein-aggregating neurodegenerative diseases was identified. We propose 17 protein targets that are likely implicated in neurodegeneration and could serve as potential targets. The human phosphatidylinositol 5-phosphatase synaptojanin-1, which has already been independently confirmed to be implicated in Parkinson’s and Alzheimer’s disease, was among those identified. Despite its involvement in PD and AD, structural aspects are currently missing at the molecular level. We present the first atomistic model of the 5-phosphatase domain of synaptojanin-1 and its binding to its substrate phosphatidylinositol 4,5-bisphosphate (PIP_2_). We determine structural information on the active site including membrane-embedded molecular dynamics simulations. Deficiency of charge within the active site of the protein is observed, which suggests that a second divalent cation is required to complete dephosphorylation of the substrate. The findings in this work shed light on the protein’s binding to phosphatidylinositol 4,5-bisphosphate (PIP_2_) and give additional insight for future targeting of the protein active site, which might be of interest in neurodegenerative diseases where synaptojanin-1 is overexpressed.

## 1 Introduction

Age-related diseases are rapidly increasing in their frequency due to longer life expectancy and can have devastating effects upon the quality of life of sufferers [1], [2]. At a cellular level, Parkinson’s disease (PD) and other neurodegenerative diseases, including Alzheimer’s disease (AD) and Amyotrophic lateral sclerosis (ALS) are linked to toxic protein aggregation [3], [4]. However, targeting these protein aggregates has not led to successful drug therapies. Small drug molecules are ineffective towards them and no new therapies for Alzheimer’s disease have been approved in the last 15 years. It is, therefore, becoming increasingly important to identify novel targets for protein-aggregating neurodegenerative diseases [5], [6].

In PD, α-synuclein is of particular importance as it is the primary aggregating protein [7], [8], [9], its gene amplifications and mutations may lead to PD [10], [11], [12]. Human neurons are complex cells with long lifespans, therefore, α-synuclein toxicity has been explored in the model eukaryotic organism, *Saccharomyces cerevisiae* (budding yeast) [13], [14], [15]. Budding yeast cells do not have a homologue to α-synuclein, therefore protein expression has been induced using a galactose inducible promoter, showing toxic aggregation in yeast which leads to cell death [16].

The abundance of biological data from various experimental sources (genomics, proteomics, metabolomics) offers unprecedented opportunities for data integration approaches for novel target identification [17]. Importantly, data integration is particularly useful in analysing networks of protein interactions and is widely used in developing understanding of how various cellular processes are altered [18]. Importantly, it can inform novel targets for atomistic studies, which is yet underutilized [19]. In this study, we employed data integration upon two complementary studies of α-synuclein toxicity: (i) genomics study by Khurana et al. and (ii) proteomics study by Melnik et al. [14], [20]. The first quantified the effect of deletion and overexpression of various proteins on the toxicity of α-synuclein in buddying yeast cells [14]. Khurana et al. compared the lifespan of yeast cells that were modified to express α-synuclein, with cells that expressed α-synuclein but had one protein deleted or overexpressed. When the deletion or overexpression of a protein significantly affected the lifetime of the cells, this protein was labelled a disease modifier: cell death enhancer or suppressor. The second dataset was collected from a proteomic analysis of the perturbation in protein wide concentrations of α-synuclein induced aggregation in buddying yeast, compared to yeast that did not express the aggregating protein [14], [20]. By integrating data from both studies, we identified 17 potential human protein targets.

Among the proteins identified to be of interest, we chose the protein polyphosphatidylinositol phosphatase INP53 for further investigation and in particular, its human homologue, synaptojanin-1 (synj1). Apart from being a cell death enhancer when deleted in α-synuclein expressing cells and simultaneously showing to be downregulated when α-synuclein was overexpressed, it has also already been independently identified that the gene coding for the protein SYNJ1, is a particular PARK locus, PARK 20 [21]. Additionally, synj1 is implicated not only in PD but also in AD [22], [23], [24], [25]. The primary substrates of synj1 are phosphoinositides (PIPs) with phosphatidylinositol 4,5-bisphosphate PIP_2_ and phosphatidylinositol 3,4,5-bisphosphate PIP_3_ being among the most important signalling lipids in membrane trafficking. An imbalance in PIPs has previously been identified to be crucial in many protein aggregating diseases, namely AD and PD [26], [27]. The imbalance of phosphoinositides is heavily correlated to malfunctions in synj1 activity, and mutations of synj1 itself are implicated in various neurodegenerative diseases [22], [23], [24], [25], [26].

Synj1 has three domains. The main catalytic inositol 5-phosphatase domain, the N-terminal Sac1 inositol phosphatase domain, and a C-terminal proline-rich domain that plays a role in protein–protein interactions related to vesicle endocytosis [9], [28]. Mutations in the Sac1 domain have already been linked to the downregulation of PIPs and malfunctions in autophagy [29].

Currently, experimentally resolved structures of the first two domains of human synj1 are unavailable. We present here the first atomistic model of the 5-phosphatase catalytic domain of the protein both in membrane-free and membrane-embedded molecular dynamics (MD) simulations. Additionally, we propose that the protein active site involves two divalent cations. It is well accepted that 5-phosphoinositide phosphatases are Mg-dependent enzymes [30], [31], with catalytic activity supported by Mg^2+^ or Mn^2+^, however inhibited by Ca^2+^ and other divalent cations [32]. This behaviour is often observed in phosphate catalytic enzymes, demonstrating apoptotic regulatory role of Ca^2+^ [33]. We suggest that one of the Mg^2+^ ions has a role in activating the water nucleophile, whereas the second Mg^2+^ stabilizes the leaving group, similar to other enzymes using a two-metal ion catalytic mechanism [34].

## 2 Methods

### 2.1 Data integration

Two data sets were used for the data integration. The first dataset was obtained by Khurana et al. [14] and was generated by comparison of the survival rate of yeast cells (*S. cerevisiae*) that were modified to express α-synuclein to cells that expressed α-synuclein but had one protein deleted or overexpressed. The proteins were labelled as either toxicity ‘Suppressor’ (S) or ‘Enhancer’ (E), based on their toxicity modulating effect on the α-synuclein expressing cells [14].

The second dataset was obtained by Melnik et al. [20]. The dataset was generated using mass spectrometry-based label-free shotgun proteomics. α-Synuclein was expressed in yeast cells (*S. cerevisiae*) by a galactose-induced promoter and the overall changes of the protein abundancies in the proteome were compared to control cells proliferating at the same time length but transformed with an empty vector (EV). Protein abundance changes were monitored at 6 h, 12 h, 18 h and 24 h after the expression of α-synuclein. Proteins which had significantly perturbed abundance at 12 h and 18 h were selected in this work (Table1 ESI and Fig. 3 ESI). Proteins perturbed at 6 h were not included as very few proteins were observed to be altered at this time suggesting that it is too early to observe the toxic effect on the cell. The results at 24 h were also omitted as the cells were dying and therefore many pathways were malfunctioning. The median ratio for the protein concentration (α-synuclein expressing cells vs. control) was the parameter used to classify the proteins as up or down regulated. The value of 1.00 was chosen as a cut-off point. If the average of the mean ratio value for the concentration of the proteins between 12 h and 18 h was above 1.00, the protein was classified ‘upregulated’, and if below 1.00, ‘downregulated’, Fig. 3 ESI.

Following this, a combined protein dataset was generated. All of the proteins that did not appear in the two initial datasets were removed. The list of proteins that had significant results in both studies were then further reduced by selecting only proteins with human homologues, using the Yeast Mine Database [35]. Finally, it was confirmed whether the protein had been previously linked to aggregation diseases using Malacards database [36].

### 2.2 Molecular dynamics simulations of Synaptojanin-1

The main catalytic 5-phosphatase domain of the human protein synj1 does not currently have an experimentally resolved structure in the protein data bank (PDB). Therefore, a homology modelling server was used (SWISS-MODEL) [37] to create the three-dimensional structure of the protein, using the amino acid sequence from the Uniprot database [38] (code: O43426). The 3D structure obtained from SWISS-MODEL was used for the MD simulations of synj1.

The 5-phosphatase domain of synj1 (residues 500–899) was modelled using the template OCRL-1 in complex with a phosphate ion (PDB code 4CMN) [30]. Residues 517–894 had a sequence identity of 36.47% to OCRL and a global model quality estimate of 0.64. A ligand with an identical phosphate head group but shortened tails was positioned manually along with the coordinating residues and water molecules, for an initial comparison of the ligand to PIP_2_. A single magnesium ion was added by visual inspection of known crystal structures based on the alignment of the conserved catalytic sites from within the 5-phosphatase family. Magnesium ion was chosen as the catalytic ion in the active site, as human 5-phosphoinositide phosphatases are Mg^2+^-dependent [30], and Zn^2+^, Ca^2+^ and other divalent ions (except for Mn^2+^) typically inhibit catalytic activity. The reference structure for positioning the PIP_2_ ligand and the magnesium ion was chosen to be the inositol polyphosphate 5-phosphatase domain (IPP5C) of SPsynaptojanin available in complex with inositol (1,4)-bisphosphate and a calcium ion (PDB code 1I9Z) [39].

All Molecular Dynamics simulations were performed by using the program NAMD [40]. The force field used in the simulations was CHARMM36 with periodic boundary conditions and to evaluate the non-bonded long-range interactions the particle mesh Ewald method was utilised with a 12 Å cutoff [41], [42]. The NPT ensemble was maintained with a Langevin thermostat (310 K) and an anisotropic Langevin piston barostat (1 atm). CHARMM-GUI was used to set up the simulation box of side length 107.4 Å; neutralise and solvate the system; and determine the charged state of all ionisable residues using a standard protocol [43], [44]. Ions randomly replaced water molecules using a Monte Carlo method to neutralise the system using 3 K^+^ ions, then additional 111 K^+^ and 111Cl^−^ ions were added to create a salt concentration of 0.15 M. Equilibration was completed using the standard CHARMM-GUI protocol [43], [44], with the addition of constraints upon the distance between the Mg^2+^ ion and: (i) the phosphate group on the fifth carbon of the inositol ring (5-P); (ii) Asp-359; (iii) Glu-92; to be approximately 3 Å [40]. 8 ns of constrained molecular dynamics simulations were completed using the constraints above, and 92 ns of non-constrained MD was run to test the stability of the membrane-free structure.

The tails of the PIP_2_ were then reinserted to the structure of the completed membrane-free simulation, and the whole structure (including the bound PIP_2_) was uploaded to the Orientation of Proteins in the Membrane (OPM) server which gave a membrane alignment for the system [45]. This alignment was then input into CHARMM-GUI to add the membrane [43], [44]. The membrane was comprised of 90% phosphatidylcholine (PC), 5% phosphatidylserine (PS) and 5% PIP_2_. To solvate the system the protein was inserted into cubic pre-equilibrated TIP3P water box of with a dimensions 127.029 Å × 127.029 Å × 129.809 Å. Ions randomly replaced water molecules using a Monte Carlo method to neutralise the system, then an additional ions (231 K^+^ and 113Cl^−^ in total) were added to create a salt concentration of 0.15 M. Six equilibration steps were conducted based on standard CHARMM-GUI protocol [43], [44]. 10 ns of constrained molecular dynamics was run, where constraints were added upon the distance of 2 Å between the Mg^2+^ ion and the following: (i) 5-P; (ii) Asp-359; (iii) Glu-92; (iv) Asn-44; the phosphate group on the fourth carbon of the inositol ring. These additional constraint for the Asn-44 residue were added to establish if additional residues were required to stop the potassium ions approaching the catalytic site. Two independent simulations of unconstrained molecular dynamics each lasting 300 ns were completed from the constrained molecular dynamics in order to obtain final structures for the Synaptojanin-PIP_2_ system.

## 3 Results and discussion

### 3.1 Data integration

**Fig. 1:**
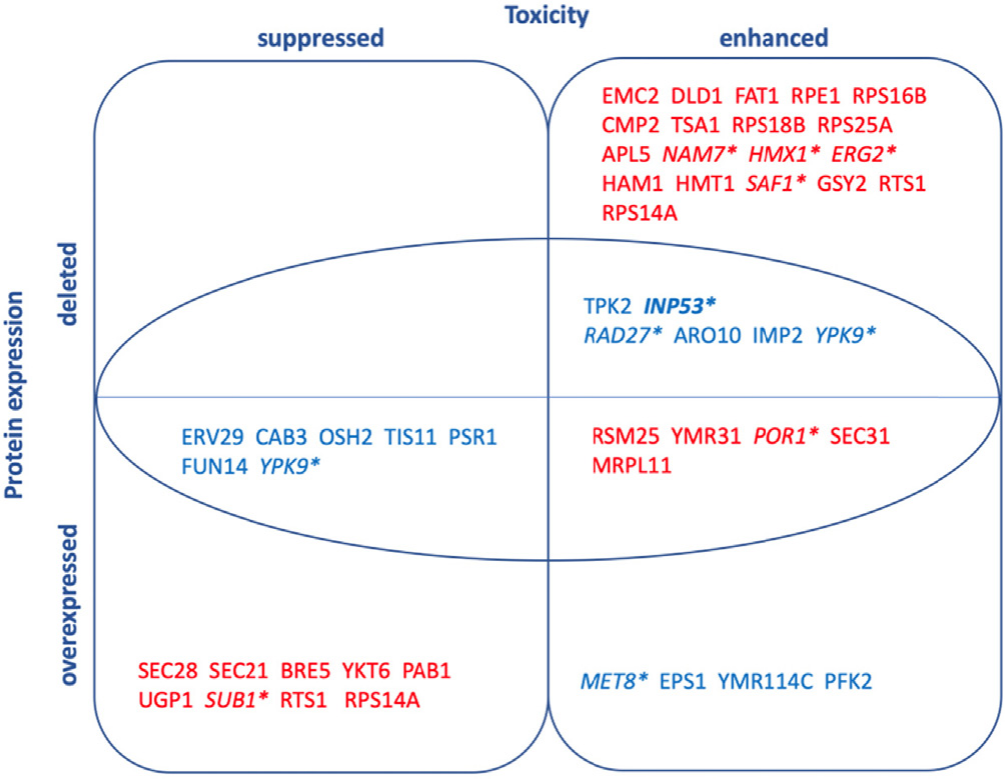
Effects of protein expression on α-synuclein cell toxicity. All proteins in the diagram have human homologues. Protein downregulation (blue) or upregulation (red) is also indicated in α-synuclein expressing cells. The circled area contains proteins identified to be proteins of interest. The human homologues of the proteins in italics with an asterisk are known to be involved in Parkinson’s or other neurodegenerative diseases [46]. INP53 (bold) is the protein chosen for further molecular dynamics modelling in this work. (For interpretation of the references to colour in this figure legend, the reader is referred to the web version of this article.)

We combined datasets from genome-wide and proteomic studies where: (i) the effects of protein deletion and overexpression on cell death was studied, and where (ii) overall protein perturbation levels were measured, in α-synuclein-expressing yeast cells. We selected those proteins that were: (i) suppressors or enhancers of cell death when deleted or overexpressed and (ii) had their concentration perturbed at 12 h and 18 h post α-synuclein expression. This data integration highlighted 62 proteins of potential interest in Parkinson’s, Alzheimer’s or other neurodegenerative diseases, based on the proteins’ toxicity modulating effect and concentration, as quantified in α-synuclein-expressing yeast cells. We then considered whether these proteins had human homologues, whether they have already been implicated in any protein aggregation diseases, and the approximate function of the protein, if known, in yeast. Upon removal of yeast specific proteins, which are not of interest to human neurodegenerative disease, the pool of proteins of interest was reduced to 47. The combined integrated data is visually represented in Fig. 1.

We further narrowed down the list of proteins to have most significance by proposing that the candidates of most interest for us would be those that either: enhance toxicity when deleted and are significantly downregulated in α-synuclein induced cells; suppress toxicity when overexpressed and their concentration is downregulated in α-synuclein induced cells; enhance toxicity when overexpressed and are significantly upregulated. These proteins are represented in Fig. 1 within the circled area.

This method of data integration highlights 17 proteins (Table1 ESI). Four of the 17 proteins (INP53, RAD27, YPK9 and POR1) have already been independently confirmed to be implicated in Parkinson’s, Alzheimer’s or other neurodegenerative diseases caused by protein aggregation [36]. Therefore, our analysis demonstrates that data integration is indeed useful in locating existing and novel protein targets that directly impact the toxicity of α-synuclein in humans, as well as in yeast, and therefore might play an important role in neurodegenerative diseases such as PD or AD.

Note that some of the proteins appear in multiple sections: YPK9, RTS1, RPS14A. For all three, cell toxicity is enhanced when the proteins are deleted, and suppressed when they are overexpressed. This is consistent with their roles as being overall needed by the cells to survive in the α-synuclein-rich environment. Interestingly, however, while RTS1, RPS14A are accordingly upregulated by the cells, YPK9 appears downregulated. YPK9 therefore has a key function, which appears to be impaired by α-synuclein overexpression, as the cells are unable to produce enough YPK9 to help cell survival.

YPK9’s human homologue, ATP13A2, is also identified by various independent measures as a key protein in PD. It is one of the PARK genes identified in human disease, PARK9, its mutations are associated with Spastic Paraplegia (SPG78), Kufor-Rakeb syndrome and neuronal ceroid lipofuscinosis [47].

The RPS14A gene’s human homologue encodes 40S ribosomal protein S14. It is a member of the ribosome, a central protein of the ribosomal protein subunit S40. It has many diverse roles and it is required for ribosome assembly and 20S pre-rRNA processing, therefore this might lead to its consistent role needed for cell survival [48], [49], [50], [51].

RTS1 is a homologue of the mammalian B′ subunit of PP2A and encodes a serine/threonine-protein phosphatase [52]. It is a central protein with several diverse roles: it is required for maintenance of septin ring organization during cytokinesis, for ring disassembly in G1 and for dephosphorylation of septin [53]. Similarly to RPS14A, a diverse regulatory function might be the reason for it being consistently beneficial for cell survival.

### 3.2 Synaptojanin-1 as potential drug target

Next, we selected synj1 to investigate further using atomistic molecular simulations. In our data integration, synj1 showed strong correlation with α-synuclein toxicity in the following ways: (i) when deleted the protein increased the rate of cell death; (ii) when α-synuclein was expressed in buddying yeast cells, the concentration of synj1 was downregulated compared to empty vector (EV) control cells that did not express α-synuclein. Based on these results the protein shows to be directly or indirectly involved in the toxicity of the aggregating protein α-synuclein. Furthermore, it is also independently confirmed by genetic analysis of PD patients’ genome that mutations of the synj1 gene have strong correlation to Parkinson’s disease [23]. Synj1 is also a PARK gene (PARK20) [21]. In addition, mutations of synj1 are also correlated with Alzheimer’s disease suggesting that it is a crucial protein in neurodegenerative diseases [25].

Synj1 does not currently have a crystallographically resolved structure except for its proline-rich domain, therefore structural studies will offer valuable insights for future drug discovery projects. We were also able to identify suitably accurate homology model template for the main catalytic 5-phosphatase domain, with an active site that is almost identical within the same phosphatase phosphoinosidtide subfamily. Other proteins of interest without available structure had lower sequence identity to template structures in the Protein Data Bank, therefore, they were less suitable candidates for molecular dynamics simulations at the time of the project start.

**Fig. 2:**
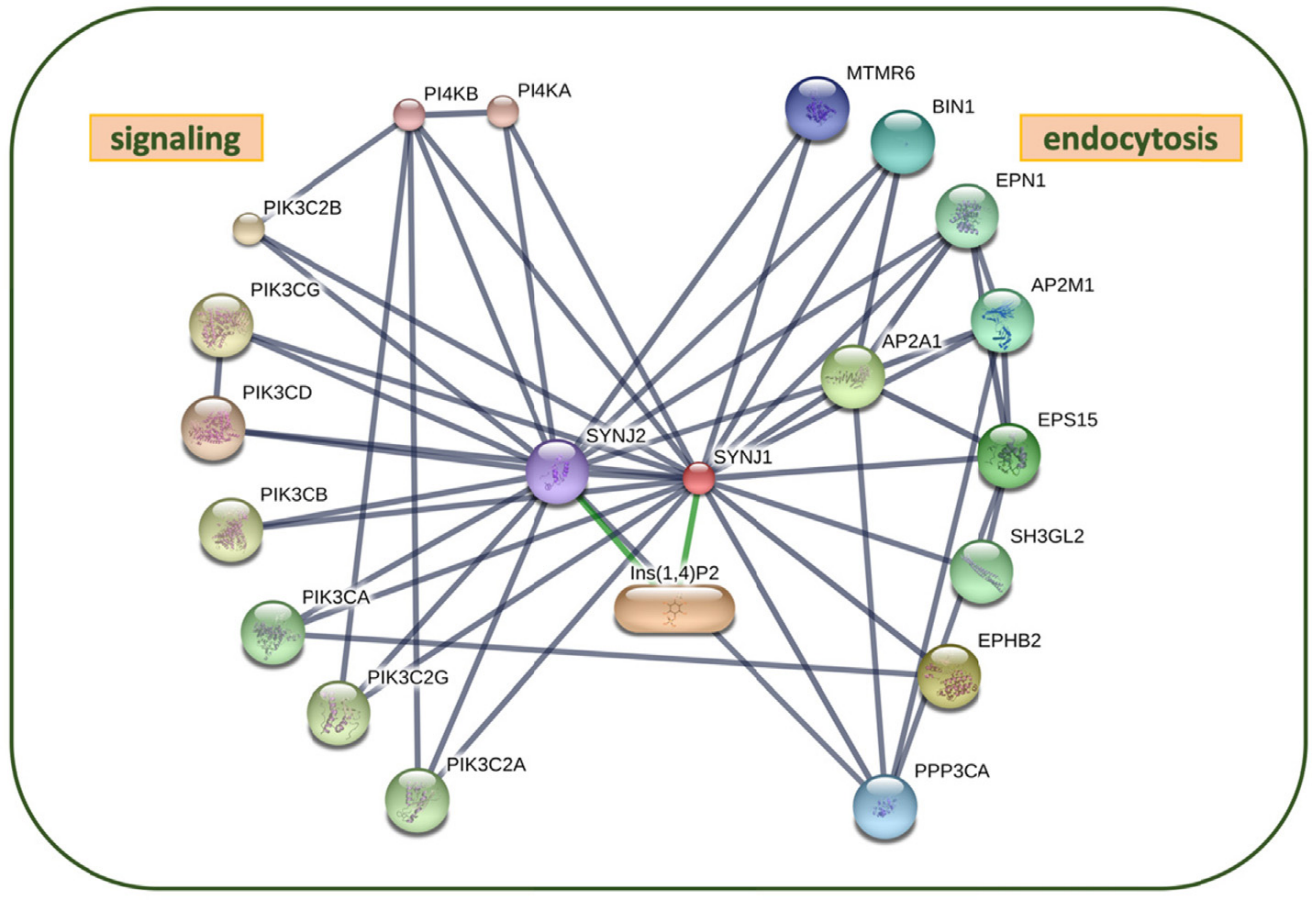
Predicted close functional partners of synj1. All proteins shown in larger nodes with cartoon have determined 3D structure, the small nodes represent proteins of unknown 3D structure. Grey lines: protein–protein interaction; green: protein-chemical. Active interaction sources: experiments, gene fusion, databases, co-occurrence, co-expression. Generated in high confidence (0.700) [54]. (For interpretation of the references to colour in this figure legend, the reader is referred to the web version of this article.)

We first determined the protein–protein interaction network of synj1 using the STRING database [54] (Fig. 2, full list of proteins and their corresponding function in Table 2 ESI). This network suggests that synj1′s primary functional partners are proteins involved indirectly or directly in synaptic vesicle endocytosis/vesicle trafficking, either through their role in PIPs regulation (signalling kinases) or in the cascade towards synaptic vesicle endocytosis. Synj1 is therefore likely mainly implicated in neurodegenerative diseases via its role in phosphatidylinositol signalling dynamics, and does not have direct effect on protein aggregation [27], [29], [55]. As part of the synaptic vesicle trafficking pathway, one of its primary catalytic functions is the dephosphorylation of the 5′P of the PIP moiety in Phosphatidylinositol 4,5-bisphosphate (PIP_2_) and Phosphatidylinositol 3,4,5-bisphosphate (PIP_3_). In this work, we have focused on the 5-phosphatase domain of the protein, which mainly dephosphorylates PIP_2_, a crucial phosphoinositide for healthy nerve function, with known effects on neurodegeneration [56], [57].

**Fig. 3:**
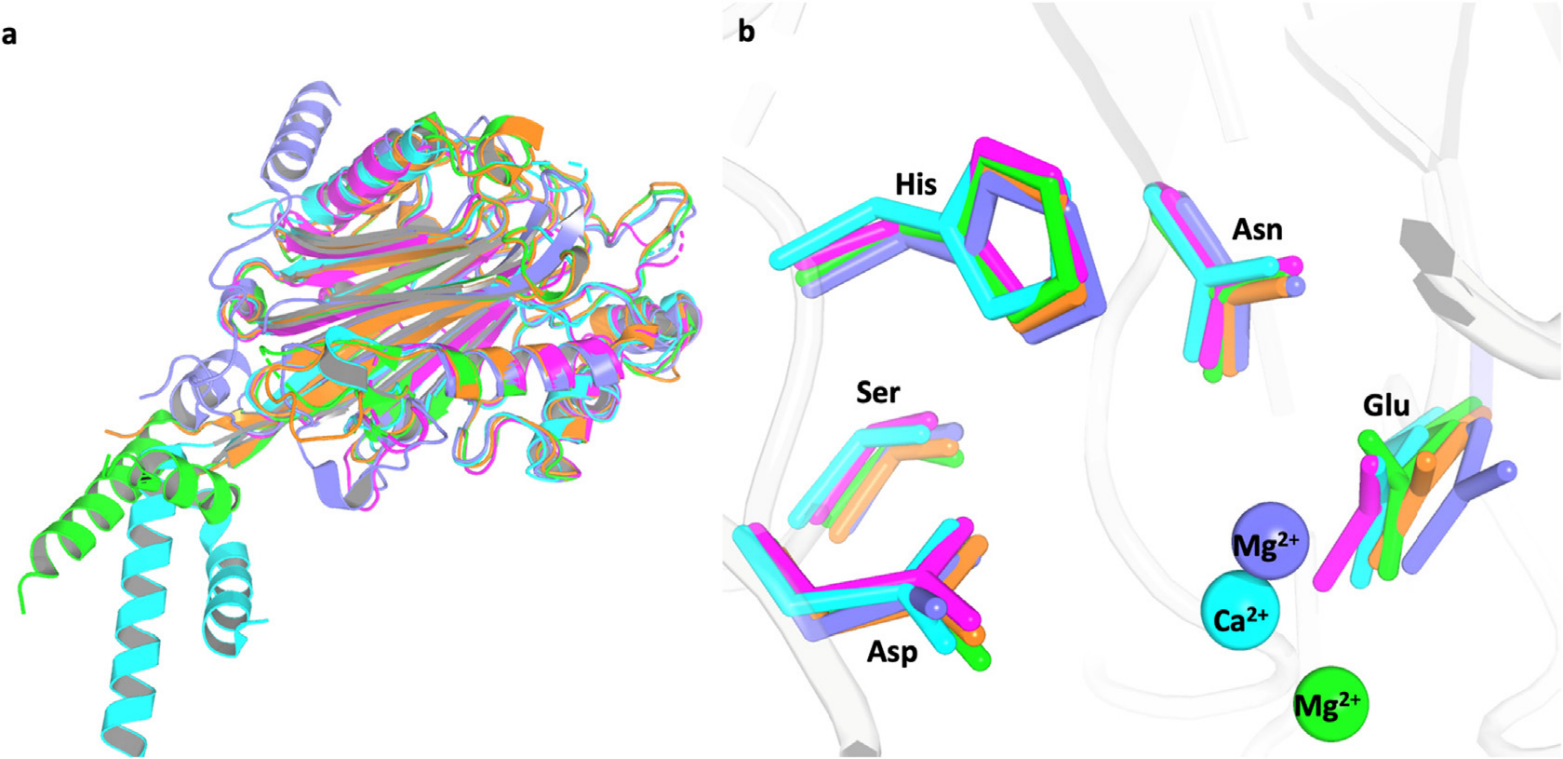
The overall conserved fold (a) and the evolutionary conserved residues (b) of 5-phosphoinositide phosphatase proteins with their code from the Protein Data Bank (PDB): yeast fission synaptojanin (1I9Z, cyan) [39], human OCRL-1 (4CMN, green) [58], human SHIP2 (4A9C, magenta) [62], human I5P2 (3MTC, orange) [58] and human Synj1 model (purple) [39], [58], [62]. (For interpretation of the references to colour in this figure legend, the reader is referred to the web version of this article.)

### 3.3 Homology modelling of Synaptojanin-1

We created a homology model of synaptojanin-1 based on the human inositol polyphosphate 5-phosphataseOCRL-1 (4CMN) [58], which has very high active site sequence identity to synaptojanin-1. There are six residues within 5 Å of the Mg^2+^ of our model that are all conserved between the model and the template protein (Fig. 1 ESI). This evidence demonstrates that the active site of synj1 can be reliably modelled based on OCRL. Additionally, charged residues are also highly conserved, which is an expected outcome for proteins with identical functionality, and additionally supports the likelihood of a reliable homology model. The sequence of synj1 (5-phosphatase domain) was also aligned and compared to other 5-phosphoinositide phosphatase proteins (Sequence in Fig. 2 ESI) [59], [60], [61]. Comparison of 5-phosphatases within the same subfamily with defined crystal structures: yeast fission synaptojanin (1I9Z) [39], human inositol polyphosphate 5-phosphatase OCRL-1 (4CMN) [58], human phosphatidylinositol 3,4,5-trisphosphate 5-phosphatase 2 SHIP2 (4A9C) [62] and human Type II inositol 1,4,5-trisphosphate 5-phosphatase I5P2 (3MTC) [58] shows the conserved overall three-dimensional fold (Fig. 3a) and evolutionary conserved residues in close proximity to the active site (Fig. 3b).

**Fig. 4:**
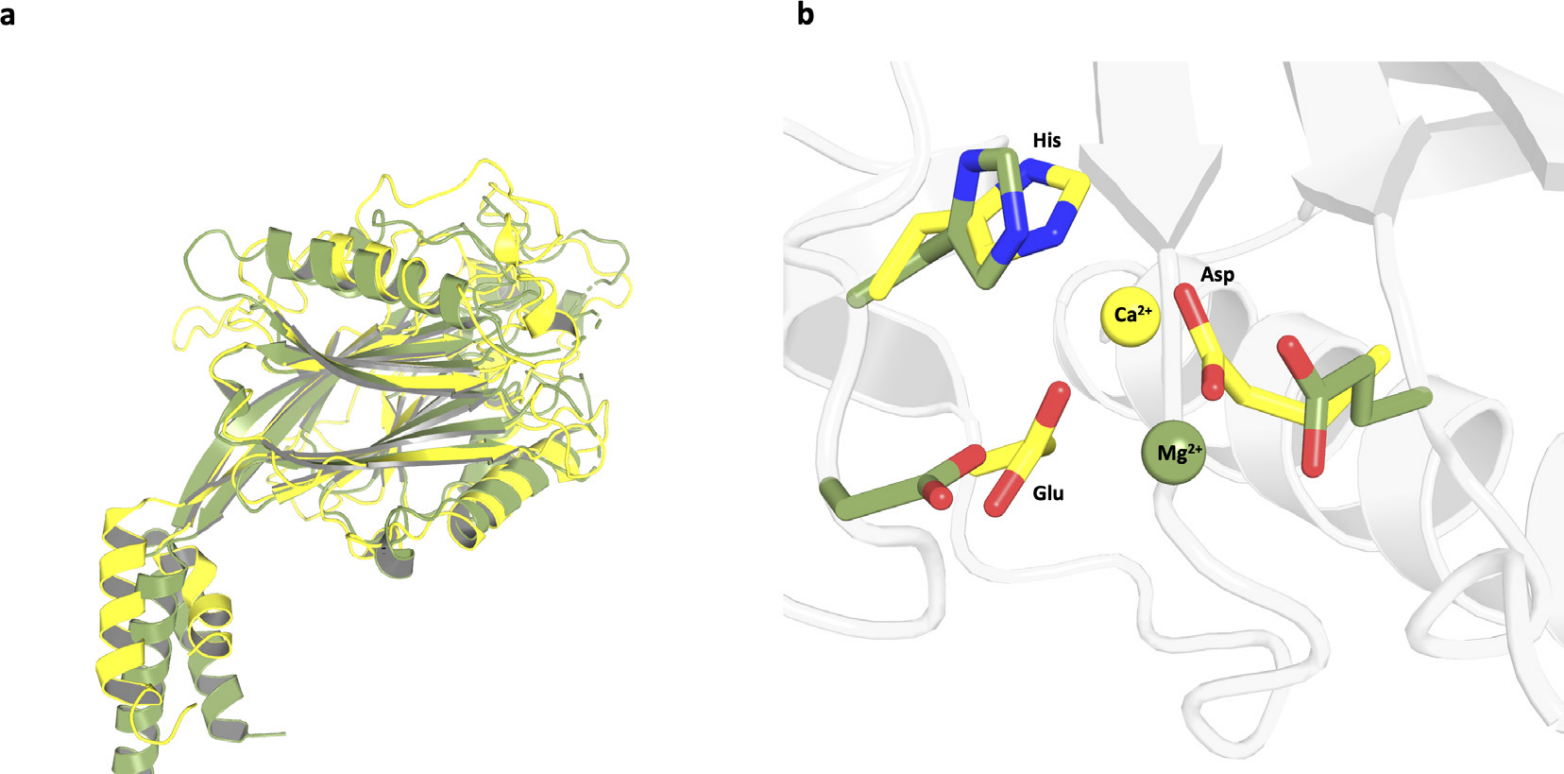
Comparison of the homology model of the 5-phosphatase domain of human synaptojanin-1 (green) with the crystal structure of fission yeast synaptojanin (yellow), (PDB: 1I9Z) [39]. Overall fold of the 5-phosphatase domain in both proteins (a), conserved residues within active site (b). (For interpretation of the references to colour in this figure legend, the reader is referred to the web version of this article.)

The completed homology model of the 5-phosphatase domain was compared to fission yeast synaptojanin (PDB:1I9Z) [39] with an overall very similar fold (Fig. 4a). It was observed that the Asp, His and Glu, the primary conserved active site residues, are located in the binding pocket for both the fission yeast synaptojanin crystal structure and the homology model of human synj1 (Fig. 4b). The conserved residues correspond to Asp-359, His-360 and Glu-92 in the homology model.

## 4 Molecular dynamics simulations

### 4.1 Membrane-free molecular dynamics simulation

**Fig. 5:**
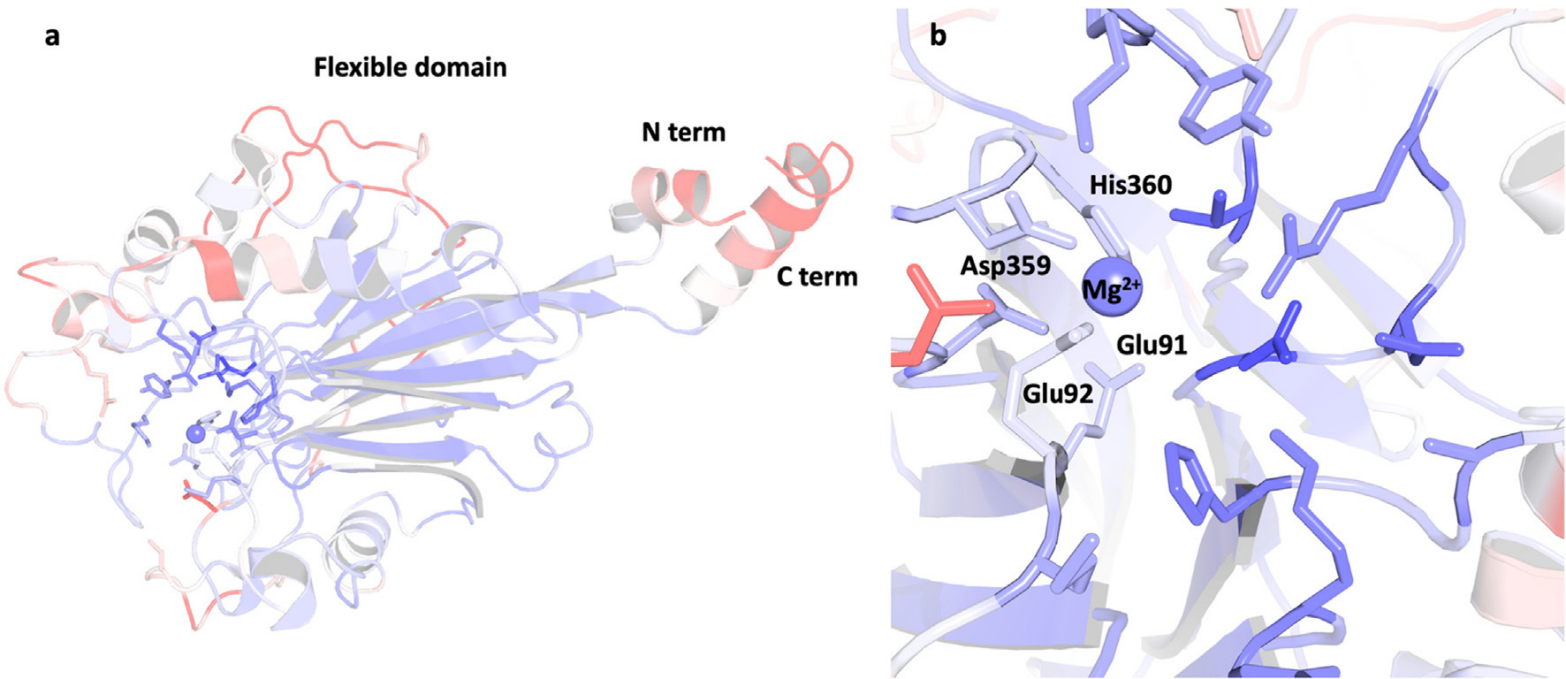
Protein coloured according to RMSD calculations of the full system (a) and catalytic domain only (b).

The flexibility of the whole protein was determined by RMSD calculations, confirming that the most flexible parts lie outside of the catalytic domain, (RMSD in Fig. 4 and Fig. 5 ESI). Fig. 5 shows the protein coloured according to the RMSD value with red signifying flexible regions and blue the more rigid parts. The flexible regions most likely belong to areas that are involved in protein–protein interactions within the synj1 or with external binding partners, as the simulations only use one domain of the protein and the interacting partners are missing from the simulations. This can be seen by the more flexible behaviour occurring at the surface of the system, mainly involving loops. This does not affect the active site or the PIP_2_ interaction as the highly flexible regions are not within significant proximity of the active site. The conformation of the synj1 active site was first probed in a membrane-free simulation and remained stable throughout the MD simulations, with an RMSD of 5 ± 1 Å. It was observed that potassium ions often appeared very close to the active site and remained there for extended periods of time. This was a surprising result, as currently the crystal structures of 5-phosphatases within the 5-phosphoinositide phosphatase family have not observed two metal ions at the active site [30], [39], [58].

### 4.2 Membrane-embedded simulations

**Fig. 6:**
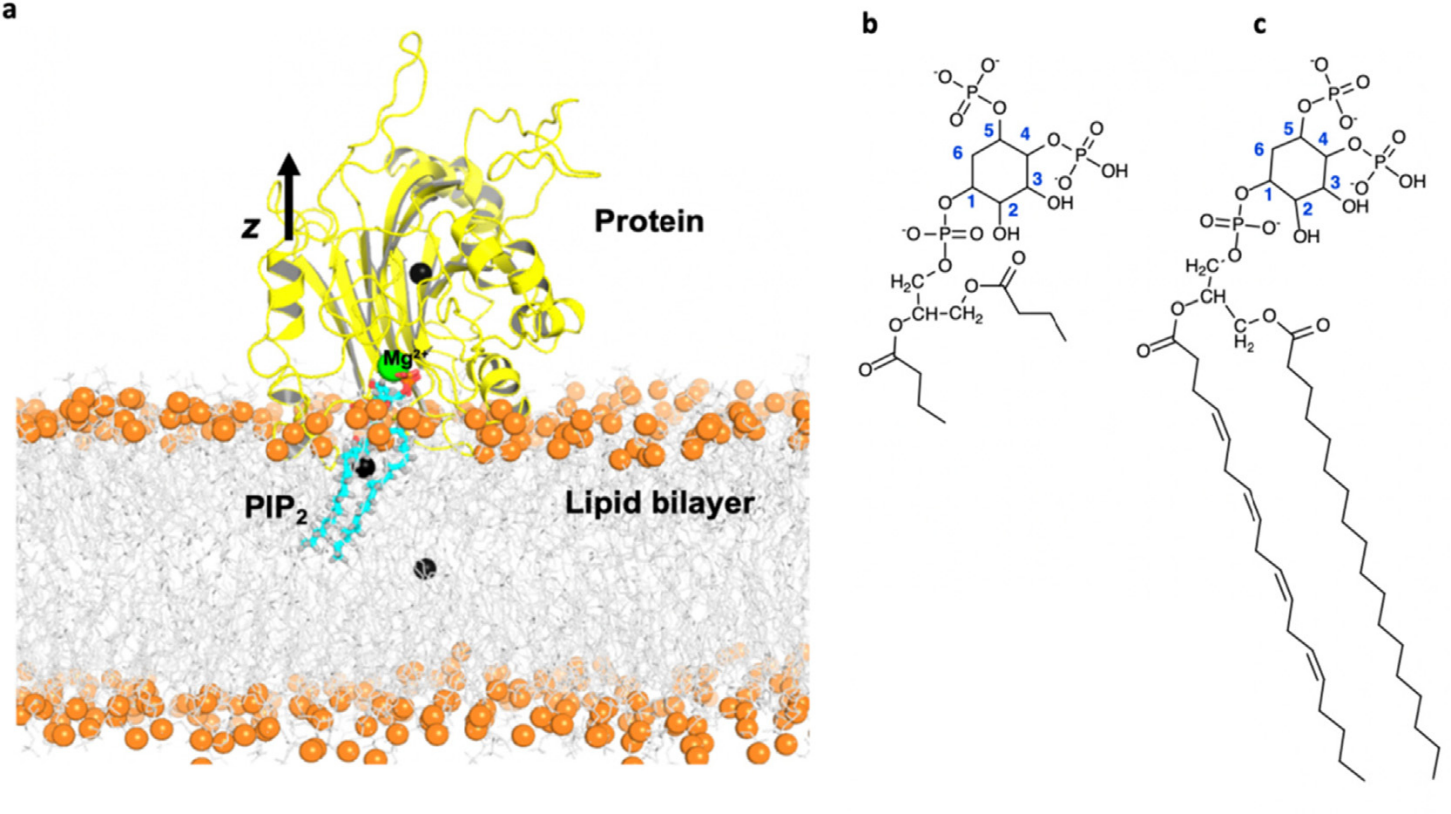
Simulation setup for the membrane-embedded simulations (a). The centres of mass (black spheres) for each component of the simulation: lipid bilayer (orange spheres and grey sticks), bound PIP_2_ ligand (blue sticks), and protein (yellow cartoon) are shown. In membrane-free simulations the ligand was modified (b) from the structure of PIP_2_ (c). (For interpretation of the references to colour in this figure legend, the reader is referred to the web version of this article.)

The unexpected presence of the potassium ions within the active site was further investigated in membrane-embedded simulations. The phospholipid bilayer consisted of 90% phosphatidylcholine (PC), 5% phosphatidylserine (PS) and 5% PIP_2_. The simulation setup of the system is shown in Fig. 6, it included the lipid bilayer, PIP_2_ ligand, protein, and the single Mg^2+^ cation. The PIP_2_ tail was indeed embedded in the membrane, and the protein located on top of the lipid bilayer allowed the phosphosugar headgroup of the PIP_2_ to bind to the synj1 active site.

**Fig. 7:**
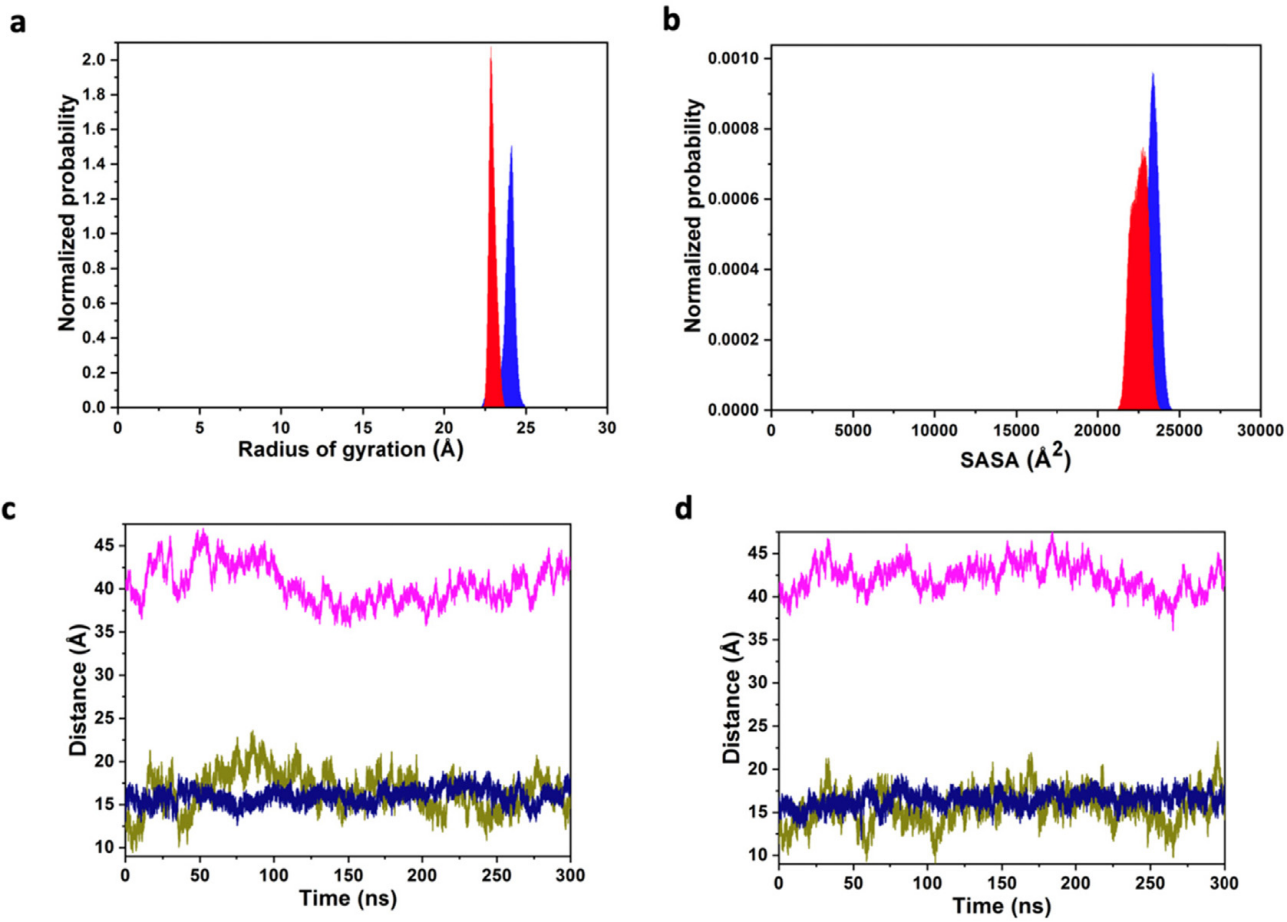
Stability measures of the synaptojanin-1 complex. (a) Radius of gyration and (b) solvent accessible surface area (SASA) distributions of the protein with a resolution of 0.03 Å and 20.0 Å, respectively. The area under each probability distribution curve is normalized to unity. (c and d) Distances between centre of mass positions projected onto the z axis for simulations 1 and 2, respectively (pink – lipid bilayer and protein; olive – lipid bilayer and PIP_2_; blue – Mg^2+^ and PIP_2_). (For interpretation of the references to colour in this figure legend, the reader is referred to the web version of this article.)

Two independent simulations were conducted over 300 ns each. The stability of the system was assessed via the radius of gyration and solvent accessible surface area of the protein (Fig. 7). The independent simulations present variation in values within a narrow range, suggesting the simulations are stable. Further analysis of the system found that the distances between both the bilayer centre of mass and the protein centre of mass, and the PIP_2_ and the magnesium ion respectively, remained stable throughout the simulation (Fig. 7). Therefore, the protein did not penetrate the bilayer, neither did the PIP_2_ ligand penetrate further into the protein.

**Fig. 8:**
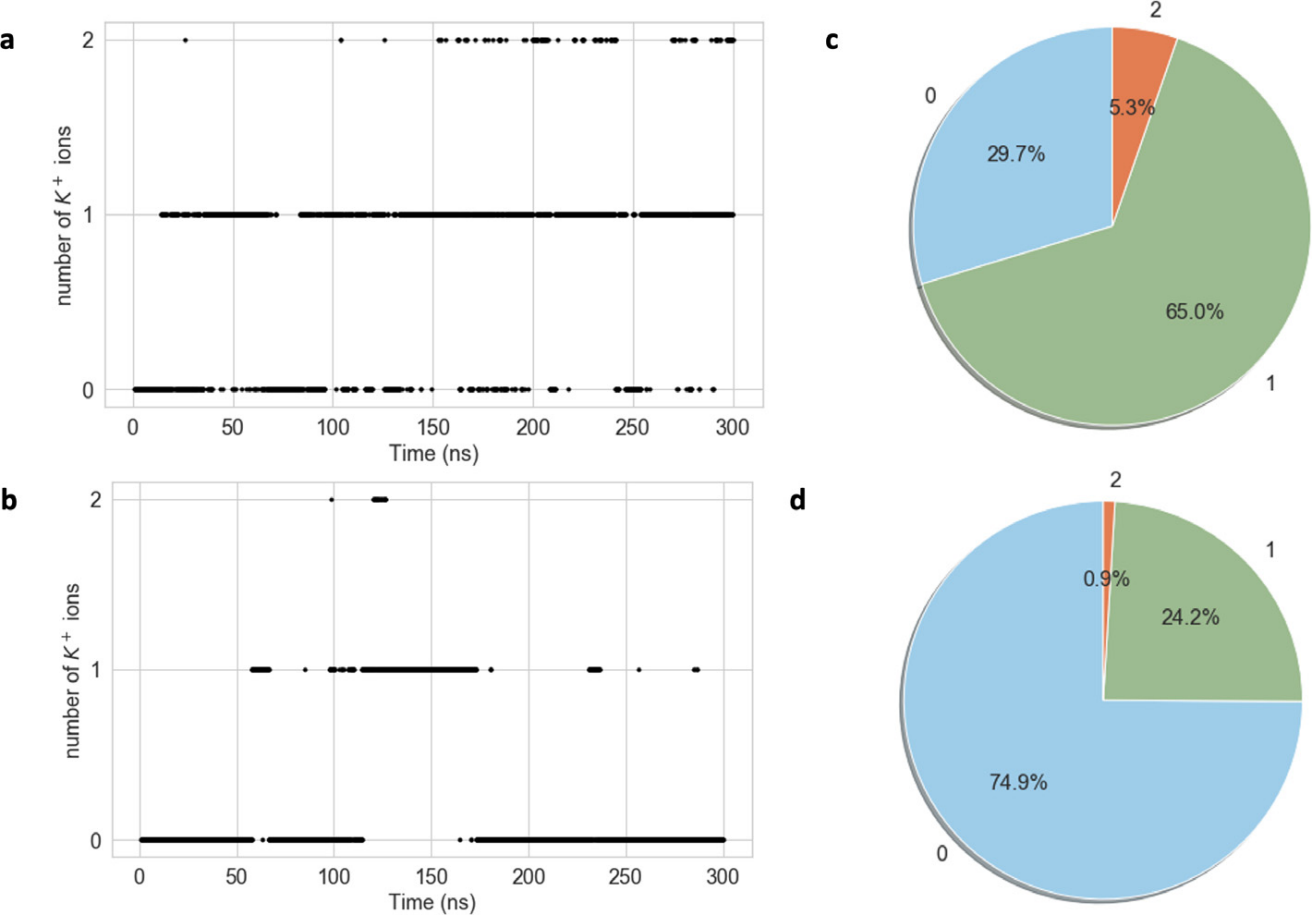
K^+^ ions within 4.5 Å of the active site during the course of simulation one (a) and simulation two (b). Percentage of 0, 1, and 2 K^+^ ions, respectively, during simulation one (c) and simulation two (d).

**Fig. 9:**
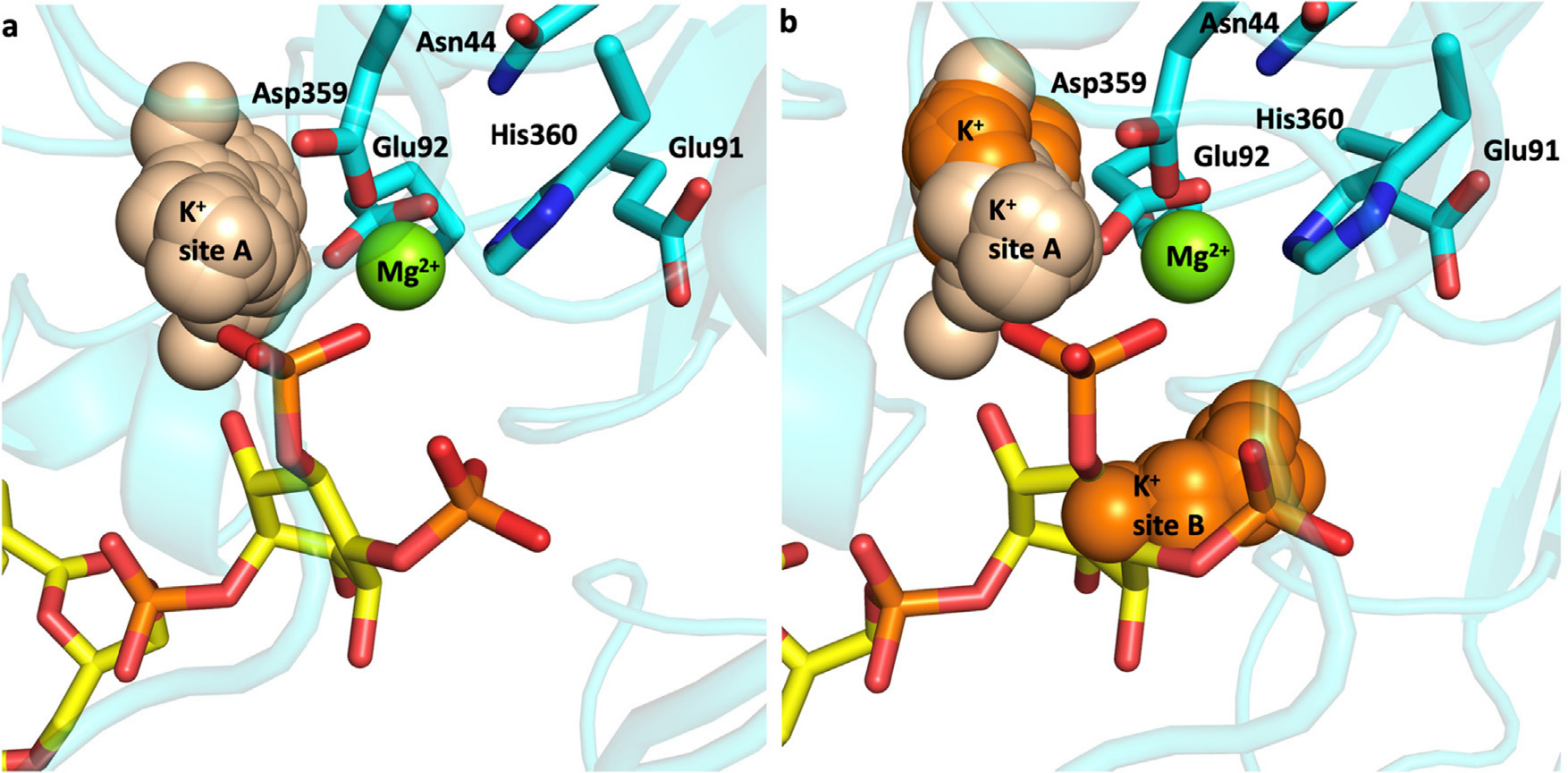
Main K^+^ binding sites A and B during simulation 1 (a) and simulation 1 and 2 (b). K^+^ population from simulation one is coloured in wheat, K^+^ population from simulation two is coloured in orange. (For interpretation of the references to colour in this figure legend, the reader is referred to the web version of this article.) The positions of the K^+^ ions are shown relative to the active site Mg^2+^-binding residues and the Mg^2+^ cation of the first simulation frame.

It was observed during both simulations that potassium ions appeared for prolonged periods of time within the active site, as quantified in Fig. 8. The active site is defined as distance of 4.5 Å or less to the 5′P atom of the PIP_2_ ligand. Consequently, if a potassium ion appears within 4.5 Å of the 5′P, it is considered to reside within the active site. There were three cases in the course of the simulation – either 0, 1 or 2 potassium ions appeared within the active site. We found that during the first and second simulations, over 70% and 25% of the time respectively, there was at least one potassium ion within 4.5 Å of 5′P. This suggests that a second positive ion is required to balance the negative charge within the binding pocket. As can be seen in Fig. 8a and b, the potassium ions approach the active site and then go away, with the number of potassium ions fluctuating constantly between 0, 1 or 2. It was observed that the localisation of the potassium cations within the active site was dependent on the orientation of the 4′phosphate group of the PIP_2_ ligand. During the second simulation the 4′phosphate group undergoes rotation, which alters the site where the K^+^ cations localise within the active site. Fig. 9 illustrates the two preferential binding sites for the potassium ions, defined here as binding site A and B. If the 4′phosphate group of the PIP_2_ remains in its original orientation for the entire 300 ns of the simulation, then the cations preferentially cluster within the same spatial region in the active site and form only one binding site (Fig. 9a). In a rotated orientation where the phosphate groups of the PIP_2_ 4′ and 5′ point in opposite directions, the K^+^ cations cluster in two locations, as depicted in Fig. 9b.

### 4.3 PIP_2_ dephosphorylation requires 2-metal-ion active site

Our MD simulations showed that the catalytic site of synj1 has a positive charge deficiency, attracting potassium ions to approach and remain within 4.5 Å of the catalytic site. Given the consistent location of these potassium ions, this suggests that synj1 functions as a two-metal ion catalytic system. Currently, all known crystal structures of phosphoinositide 5-phosphatases have been resolved with only a single Mg^2+^ ion bound in the active site [30], [39], [58]. However, our simulations used the catalytically active ligand, PIP_2_, as opposed to the inactive protein–ligand complexes with synthetic derivatives resolved structurally. Furthermore, we also included the membrane environment not present in these crystallographic structures. We found that PIP_2_ does not significantly change its conformation with respect to the protein or the lipids but rather the potassium ions do approach the highly conserved catalytic residues, further supporting the suggestion that one magnesium ion may be insufficient. The occupancy of the potassium binding pocket within the active site continuously changes between 0, 1 and 2 ions during the course of the simulations, highlighting the openness of the binding site, which is likely only stable once the catalytic complex is correctly assembled. This could provide an explanation for the lack of crystallographic observation of the second metal ion.

Independently from our current work, various studies suggest that 5-phosphatases operate via the same catalytic mechanism as Mg^2+^-dependent DNA restriction endonucleases, including the members DNase I and the apurinic/apyrimidinic base excision repair endonuclease Ape1 [63], [64]. The conserved catalytic mechanism of the same 5-phosphate-type cleavage reaction is supported by evolutionarily strongly conserved amino acid sequence motifs within the active site (Fig. 6, ESI) [64]. Various endonuclease structures have been resolved with 2 cations within the active site, further suggesting that synj1 might also operate as a two-ion catalytic system [34], [65]. Previous MD simulations of Ape1 also suggested the possible transient transfer between the two metal ion locations, termed “moving metal mechanism” [66], however, we do not observe evidence for such a mechanism in our simulations.

Additionally, biochemical experiments using various Mg^2+^ and Ca^2+^ concentrations also support the two-metal ion catalytic mechanism. Two metal-binding sites, each with a distinct binding affinity, are expected to yield biphasic inhibition curves when titrated with a non-productive metal. These bimodal effects were observed for APE1 further supporting that two metal ions are required for the catalytic reaction [34].

### 4.4 Synaptojanin-1 binding to PIP_2_ and the potential for drug therapy

The importance of understanding the binding mechanism between synaptojanin-1 and the phosphatidylinositol phosphates has been already established. This understanding creates the potential for a new drug target. The MD simulations discussed in this work have achieved new insight into this binding process. It was shown that the PIP_2_ bound to the synaptojanin-1 complex is stable and relatively open as the protein needs to also interact with the membrane surface to bind to PIP_2_. This opens the possibility of drug molecules potentially interfering with the binding process, which could be used to decrease the activity of the protein in neurodegenerative diseases where upregulation increases the pathogenicity of the disease, for example in Alzheimer’s disease. The decreased expression of synaptojanin-1 in AD has been shown to be protective and aids in amyloid-beta clearance [9], [22]. Any drug created would need to be carefully administered as uncontrolled downregulation of the protein can also be harmful, as seen in our data integration results. The drug target would also need to interact preferentially with synj1 over the other phosphatidylinositol 5-phosphatases, all of which have very similar catalytic sites. Due to this, it may be worthwhile investigating whether targeting other regions of synj1 may be preferential. Alternatively, a drug target may bind to a synj1-specific surface that interferes with the membrane interactions, preventing PIP_2_ binding.

### 4.5 Using yeast to predict key proteins in neurodegenerative diseases

The utilisation of yeast to predict the most important proteins in neurodegenerative diseases in humans has been found to have many benefits. As yeast is a much simpler cell than a neuron and is a single-celled organism, it significantly reduces the complexity of the problem. It also has a much shorter lifespan making it easier to study and collect sufficient data upon [67]. In humans, we generally use post-mortem samples or positron emission tomography (PET), which are potentially not very effective methods for identifying early markers and causative processes of a disease, as they are not single cell methods [68]. Ideally, preferred therapies intervene before significant cell death, cognitive decline and bradykinesia occur, enabling a higher quality of life for patients. Many proteins that have been discovered to have an effect on human disease progression are identified by mutations that cause harmful effects in the protein, and subsequently increase the likelihood or speed of disease progression [69]. Using mutations to identify proteins related to disease while useful, does not necessarily aid in understanding the sporadic disease, or general disease pathway. It is possible to identify proteins that suppress disease progression in wild type cells, but when mutated are unable to perform their function and lead to increased disease progression, as well as those that are already actively exacerbating the disease in wild type cells. Using yeast where high throughput genomic and proteomic studies are regularly conducted, it is possible to combine multiple datasets in the hope to provide more insight into the effect of the non-mutated proteins on neurodegenerative diseases’ progression [13], [14], [16]. However, arguably, the most significant drawback of this method is that neurodegenerative diseases are often developing at the synapse, which is not present in yeast. For this reason, any yeast-based method, including the data integration found in this work, cannot identify any neuron-specific proteins or pathways but rather generic cell pathways that are conserved in both humans and yeast, and so invariably they will be proteins that are highly conserved across all eukaryotes. This is the underlying reason why the 17 candidate proteins found are primarily involved in processes or organelles that are ubiquitous across eukaryotes; with many linked to the mitochondria and its associated processes. Data integration is still a very powerful tool as it has been possible not only to investigate the effect of α-synuclein aggregation upon protein concentrations in the cell, but also how these perturbations in protein concentration may be altering the toxicity of aggregation [14], [20].

## 5 Conclusions

The wealth of biological information currently being produced requires new approaches to interpret and utilise the data so that we maximally filter useful information. Data integration is one possibility that could enable us to reuse data that is currently under-utilised. This is particularly beneficial as it does not require conducting more experiments to gain more information. Using this principle of data integration, two large scale studies of α-synuclein induction in budding yeast were analysed and used to identify 17 proteins that could be of interest in human PD and AD. Most of these 17 proteins were found to be related to human diseases, either directly or indirectly.

Among those, we chose to investigate further the 5-phosphatase domain of the regulatory lipid phosphatase synaptojanin-1. By dysregulation of various PIPs, the malfunction of synj1 is linked to the decrease of cell health and increase of proteomic stress. Synaptojanin-1 dephosphorylates the phosphatidylinositol PIP_2_ at the synapse membrane. The catalytic function is carried out through an interaction with an essential coordinating magnesium cofactor. Through all-atom MD simulations including the membrane and the PIP_2_-bound protein, we observed that the proposed catalytic site was stable, but potassium ions persistently approached the binding pocket. This suggests that another positive charge is required for a catalytically active complex. Therefore, we propose that synj1 is likely using a two-metal ion catalytic mechanism for its phosphatase function. Current human phosphoinositide 5-phosphatases are all resolved crystallographically with only a single metal ion at the active site. Future work on synj1 could confirm our results via high-resolution crystal structures, or by biochemical measurements on the effects of mutations at the catalytic site or using Mg^2+^ concentration-dependent catalytic rate measurements. This would be particularly beneficial in future targeting of the active site.

Our work identifies potential novel targets for α-synuclein aggregating diseases. Furthermore, it provides the first atomistic investigation of the human synj1 main 5-phosphatase catalytic domain. Our novel structural information could potentially enable the design of a small molecule inhibitor that could prevent or destabilise PIP_2_ binding, leading to a novel avenue for disease therapy where decreasing synj1 activity can be beneficial.

## CRediT authorship contribution statement

**Kirsten Jenkins:** Conceptualization, Methodology, Data curation, Visualization, Writing - original draft. **Teodora Mateeva:** Methodology, Data curation, Visualization, Writing - original draft, Writing - review & editing. **István Szabó:** Methodology, Visualization. **Andre Melnik:** Methodology, Data curation. **Paola Picotti:** Conceptualization, Methodology, Writing - review & editing, Supervision. **Attila Csikász-Nagy:** Conceptualization, Methodology, Writing - original draft, Writing - review & editing, Supervision. **Edina Rosta:** Conceptualization, Methodology, Writing - original draft, Supervision, Writing - review & editing, Visualization.
