# Supplementary material for: Combining data integration and molecular dynamics for target identification in α-Synuclein-aggregating neurodegenerative diseases: Structural insights on Synaptojanin-1 (Synj1)
Source: Comput Struct Biotechnol J. 2020 Apr 22;18:1032–42. doi: 10.1016/j.csbj.2020.04.010 (PMC7215115; doi:10.1016/j.csbj.2020.04.010)
Supplement: Supplementary data 2 [file mmc2.pdf]

Electronic Supplementary Information (ESI)

Combining data integration and molecular dynamics for target identification in ∝-synuclein-aggregating neurodegenerative diseases: Structural insights on Synj1

Kirsten Jenkins,[a] Teodora Mateeva,[b] István Szabó,[b] Andre Melnik,[c] Paola Picotti,[c] Attila Csikász-Nagy,[a,d] Edina Rosta[b*]

[a]Randall Division of Cell and Molecular Biophysics, Institute for Mathematical and Molecular Biomedicine, King's College London, London SE1 1UL, UK

[b]Department of Chemistry, King's College London, London SE1 1DB, UK

[c]Institute of Biochemistry, Department of Biology, ETH Zurich, CH-8093 Zurich, Switzerland

[d]Faculty of Information Technology and Bionics, Pázmány Péter Catholic University, 1083 Budapest, Hungary

*Corresponding author:

E-mail: edina.rosta@kcl.ac.uk

Electronic Supplementary Tables

Table 1 ESI. Proteins chosen to be of interest in this work. The corresponding human homologue of the yeast protein is shown in the second column [1]. Third and fourth column show the disease modulating effect of the protein on ∝-synuclein toxicity, as found in the Khurana *et. al* study [2]. Fifth and sixth column show the median ratio for the protein concentration between ∝-synuclein expressing cells and control empty vector (EV) cells. The final column shows the average of the median ratio value (∝-synuclein expressing vs. control at 12h and 18h) from the Melnik *et. al* study [3]. Values coloured in red signify upregulated proteins, blue - downregulated.

Yeast Protein	Human Homologue	Deletion Modulator	Overexpression Modulator	Median ratio value for the protein c. at 12h	Median ratio value for the protein c. at 8h	Average of median ratio value between 12h and 18h
ERV29	SURF4		Suppressor	0.9487	0.7617	0.8552
CAB3	PPCDC		Suppressor	0.9913	0.2275	0.6094
OSH2	OSBP		Suppressor	0.7693	0.7696	0.7695
TIS11	ZFP36		Suppressor	0.5446	1.0161	0.7803
PSR1	CTDSP2		Suppressor	0.7297	0.3574	0.5436
FUN14	FUNDC1		Suppressor	0.5588	0.9406	0.7497
YPK9	ATP13A2	Enhancer	Suppressor	0.5094	0.6475	0.5785
TPK2	PRKACB	Enhancer		0.6505	0.6821	0.6663
INP53	SYNJ1	Enhancer		0.7353	1.0839	0.9096
RAD27	FEN1	Enhancer		0.8404	0.5271	0.6837
ARO10	ILVBL	Enhancer		0.7201	0.9930	0.8566
IMP2	IMPP2L	Enhancer		0.3738	0.3737	0.3738
RSM25	MRPS23		Enhancer	1.6853	1.5705	1.6279
YMR31	MRPS36		Enhancer	1.6649	1.7560	1.7105
POR1	VDAC1		Enhancer	1.1816	1.5695	1.3756
SEC31	SEC31B		Enhancer	1.3086	1.1884	1.2485
MRPL11	MRPL10		Enhancer	1.6600	1.6863	1.6732

Table 2 ESI. Close functional partners to synaptojanin-1. Generated using STITCH [4].

Protein name	Function
**PIK3CA**	Phosphatidylinositol-4,5-bisphosphate 3-kinase, **catalytic subunit alpha**; Phosphoinositide-3-kinase (PI3K) that phosphorylates PtdIns (Phosphatidylinositol), PtdIns4P (Phosphatidylinositol 4- phosphate) and PtdIns(4,5)P2 (Phosphatidylinositol 4,5- bisphosphate) to generate phosphatidylinositol 3,4,5-trisphosphate (PIP_3). PIP_3 plays a key role by recruiting PH domain-containing proteins to the membrane, including AKT1 and PDPK1, activating signalling cascades involved in cell growth, survival, proliferation, motility and morphology.
**PIK3CB**	Phosphatidylinositol-4,5-bisphosphate 3-kinase, **catalytic subunit beta**; Phosphoinositide-3-kinase (PI3K) that phosphorylates PtdIns (Phosphatidylinositol), PtdIns4P (Phosphatidylinositol 4- phosphate) and PtdIns(4,5)P2 (Phosphatidylinositol 4,5- bisphosphate) to generate phosphatidylinositol 3,4,5-trisphosphate (PIP3). PIP3 plays a key role by recruiting PH domain-containing proteins to the membrane, including AKT1 and PDPK1, activating signalling cascades involved in cell growth, survival, proliferation, motility and morphology.
**PIK3CD**	Phosphatidylinositol-4,5-bisphosphate 3-kinase, **catalytic subunit delta**; Phosphoinositide-3-kinase (PI3K) that phosphorylates PftdIns(4,5)P2 (Phosphatidylinositol 4,5-bisphosphate) to generate phosphatidylinositol 3,4,5-trisphosphate (PIP_3). PIP_3 plays a key role by recruiting PH domain-containing proteins to the membrane, including AKT1 and PDPK1, activating signalling cascades involved in cell growth, survival, proliferation, motility and morphology. Mediates immune responses. Plays a role in B-cell development, proliferation, migration, and function.
**PIK3CG**	Phosphatidylinositol-4,5-bisphosphate 3-kinase, **catalytic subunit gamma**; Phosphoinositide-3-kinase (PI3K) that phosphorylates PtdIns(4,5)P2 (Phosphatidylinositol 4,5-bisphosphate) to generate phosphatidylinositol 3,4,5-trisphosphate (PIP_3). PIP_3 plays a key role by recruiting PH domain-containing proteins to the membrane, including AKT1 and PDPK1, activating signalling cascades involved in cell growth, survival, proliferation, motility and morphology. Links G-protein coupled receptor activation to PIP_3 production.
**EPHB2**	EPH receptor B2; Receptor tyrosine kinase which binds promiscuously transmembrane ephrin-B family ligands residing on adjacent cells, leading to contact-dependent bidirectional signalling into neighbouring cells. The signalling pathway downstream of the receptor is referred to as forward signalling while the signalling pathway downstream of the ephrin ligand is referred to as reverse signalling. Functions in axon guidance during development. Involved in the guidance of commissural axons, that form a major interhemispheric connection between the 2 temporal lobes of the cerebral cortex.
**SH3GL2**	Endophilin-A3; Implicated in endocytosis. May recruit other proteins to membranes with high curvature.
**EPS15**	Epidermal growth factor receptor substrate 15; Involved in cell growth regulation. May be involved in the regulation of mitogenic signals and control of cell proliferation. Involved in the internalization of ligand-inducible receptors of the receptor tyrosine kinase (RTK) type, in particular EGFR. Plays a role in the assembly of clathrin-coated pits (CCPs). Acts as a clathrin adapter required for post-Golgi trafficking. Seems to be involved in CCPs maturation including invagination or

	budding. Involved in endocytosis of integrin beta-1 (ITGB1) and transferrin receptor (TFR).
**EPN1**	Epsin 1; Binds to membranes enriched in phosphatidylinositol 4,5- bisphosphate (PtdIns(4,5)P2). Modifies membrane curvature and facilitates the formation of clathrin-coated invaginations.
**BIN1**	Myc box-dependent-interacting protein 1; May be involved in regulation of synaptic vesicle endocytosis. May act as a tumor suppressor and inhibits malignant cell transformation.
**AP2A1**	AP-2 complex subunit alpha-1; Component of the adaptor protein complex 2 (AP-2). Adaptor protein complexes function in protein transport via transport vesicles in different membrane traffic pathways. Adaptor protein complexes are vesicle coat components and appear to be involved in cargo selection and vesicle formation. AP-2 is involved in clathrin-dependent endocytosis in which cargo proteins are incorporated into vesicles surrounded by clathrin (clathrin-coated vesicles, CCVs) which are destined for fusion with the early endosome.
**AP2M1**	Adaptor-related protein complex 2, mu 1 subunit; Component of the adaptor protein complex 2 (AP-2). Adaptor protein complexes function in protein transport via transport vesicles in different membrane traffic pathways. Adaptor protein complexes are vesicle coat components and appear to be involved in cargo selection and vesicle formation. AP-2 is involved in clathrin-dependent endocytosis in which cargo proteins are incorporated into vesicles surrounded by clathrin (clathrin- coated vesicles, CCVs) which are destined for fusion with the early endosome.
**MTMR6**	Myotubularin related protein 6; Phosphatase that acts on lipids with a phosphoinositol headgroup. Acts as a negative regulator of KCNN4/KCa3.1 channel activity in CD4+ T-cells possibly by decreasing intracellular levels of phosphatidylinositol 3 phosphatase. Negatively regulates proliferation of reactivated CD4+ T-cells.
**SYNJ2**	Synaptojanin 2; Inositol 5-phosphatase which may be involved in distinct membrane trafficking and signal transduction pathways. May mediate the inhibitory effect of Rac1 on endocytosis.
**PI4KB**	Phosphatidylinositol 4-kinase, catalytic, **beta**; Phosphorylates phosphatidylinositol (PI) in the first committed step in the production of the second messenger inositol- 1,4,5,-trisphosphate (PIP). May regulate Golgi disintegration/reorganization during mitosis, possibly via its phosphorylation.
**PI4KA**	Phosphatidylinositol 4-kinase, catalytic, **alpha**; Acts on phosphatidylinositol (PtdIns) in the first committed step in the production of the second messenger inositol- 1,4,5,-trisphosphate.
**PIK3C2B**	Phosphatidylinositol-4-phosphate 3-kinase, catalytic subunit type 2 beta; Phosphorylates PtdIns and PtdIns4P with a preference for PtdIns. Does not phosphorylate PtdIns(4,5)P2. May be involved in EGF and PDGF signaling cascades.
**PIK3C2G**	Phosphatidylinositol-4-phosphate 3-kinase, catalytic subunit type 2 gamma; Generates phosphatidylinositol 3-phosphate (PtdIns3P) and phosphatidylinositol 3,4-bisphosphate (PtdIns(3,4)P2) that act as second messengers.
**PIK3C2A**	Phosphatidylinositol-4-phosphate 3-kinase, catalytic subunit type 2 alpha; Generates phosphatidylinositol 3-phosphate (PtdIns3P) and phosphatidylinositol 3,4-bisphosphate (PtdIns(3,4)P2) that act as second messengers.
**PPP3CA**	Protein phosphatase 3, catalytic subunit, alpha isozyme; Calcium-dependent, calmodulin-stimulated protein phosphatase. This subunit may have a role in the calmodulin activation of calcineurin. Dephosphorylates DNM1L, HSPB1 and SSH1.

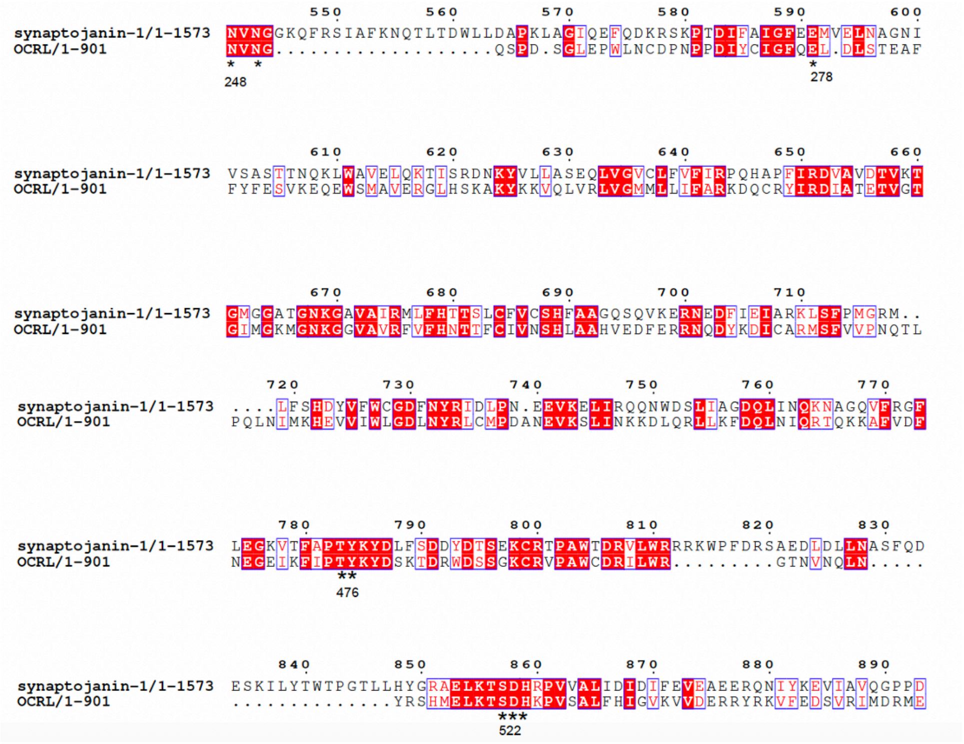

Fig. 1 ESI. The 5-phosphatase domain of human Synj1 (Synaptojanin-1, uniport code: O43426) and OCRL (Inositol polyphosphate 5-phosphatase, uniport code: Q01968) are aligned. The conserved residues in the active site are marked with an asterisk. The numbering is based on synj1. The corresponding numbering of the active site residues in the OCRL protein are shown below.

Sequence analysis was performed on proteins within the 5-phosphoinositide phosphatase family (OCRL, I5P2, SYNJ1, SHIP2) [5–8] using the algorithm ClustalW and Muscle [9,10] (as implemented in Jalview) [11] and visualised with Espript 3.0 [12]. The important conserved residues within the active site are marked with a red asterisk. The Mg cation-coordinating residues, corresponding to His 360, Asp 359 and Glu 92 in the homology model, are highly conserved in this family and are marked with asterisk. This sequence alignment explores only the 5-phosphatase domain of the proteins.

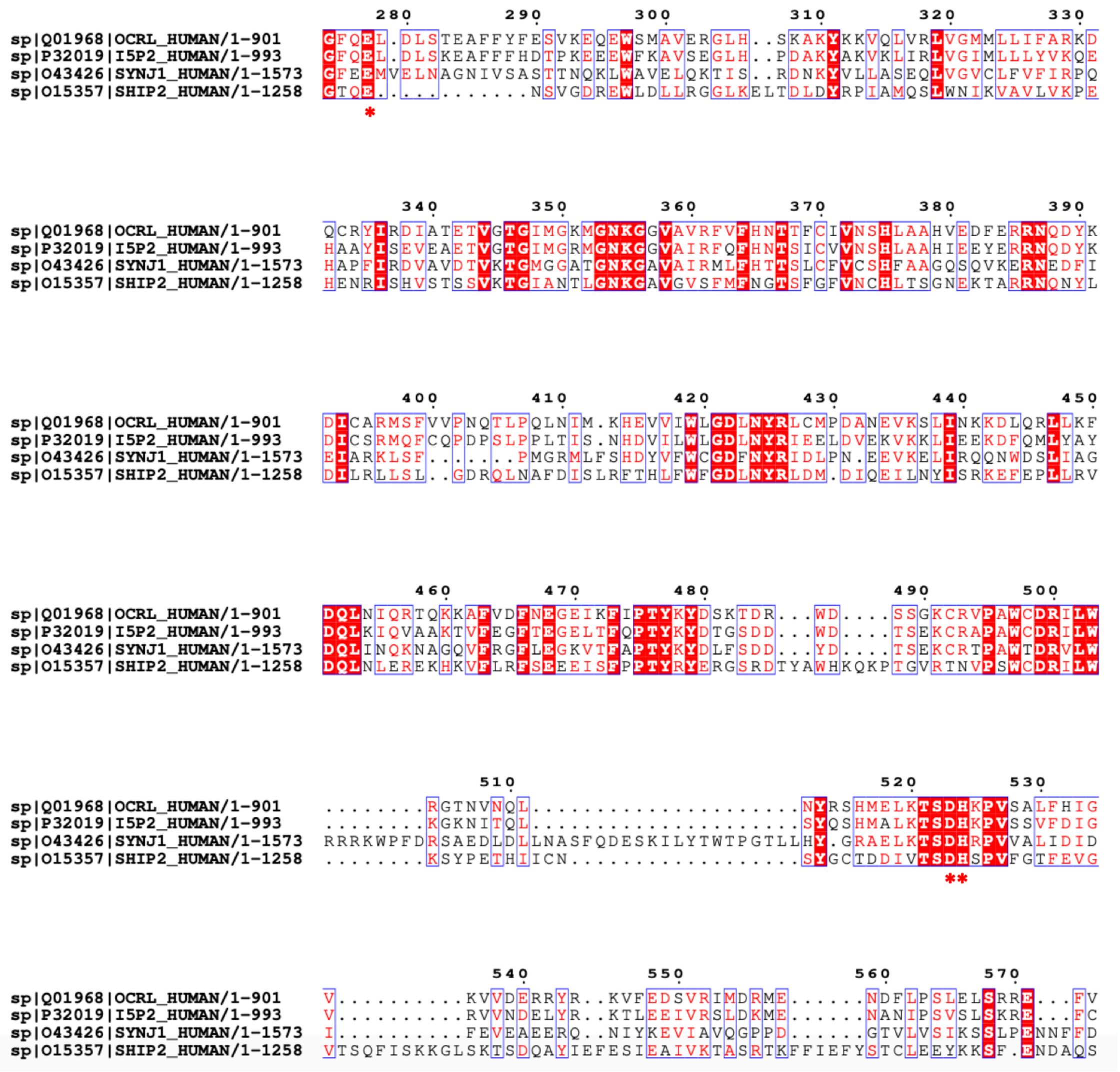

Fig. 2 ESI. Amino acid sequence comparisons of the 5-phosphatase domain of human 5-phosphoinositide phosphatases. Alignments were performed using the Clustal W and Muscle algorithm as implemented in Jalview and visualisation was done with Espript 3.0 [9–12]. Residue numbering is based on OCRL. The conserved residues in the active site Asp (**D**), His (**H**) and Glu (**E**) found for all 5-phosphatases (SHIP2, Synj1, OCRL, INPPB5) [5,6,8] are marked with an asterisk. These are Mg-coordinating and correspond to His-360, Asp-359 and Glu-92 in the homology model. All sequences are obtained from Uniprot, using the Homo sapiens sequence [13].

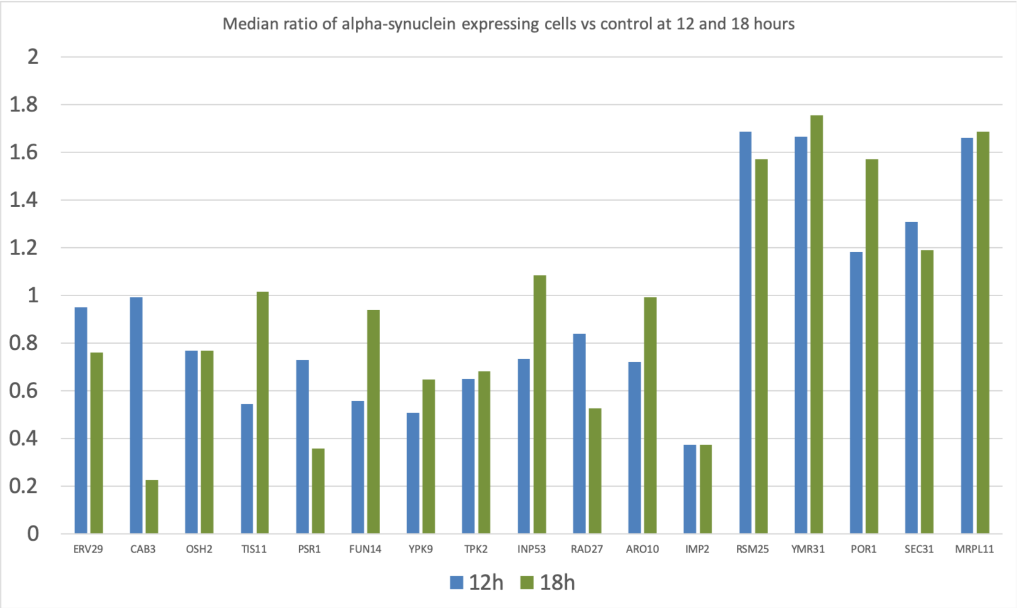

Fig. 3 ESI. Median ratio of protein concentration in ∝-synuclein expressing cells vs. control at 12h and 18h. Proteins which have a median ratio value of above 1 (averaged between 12h and 18 h), have been defined as 'upregulated', proteins with a value below 1 have been defined as downregulated in ∝-synuclein expressing cells.

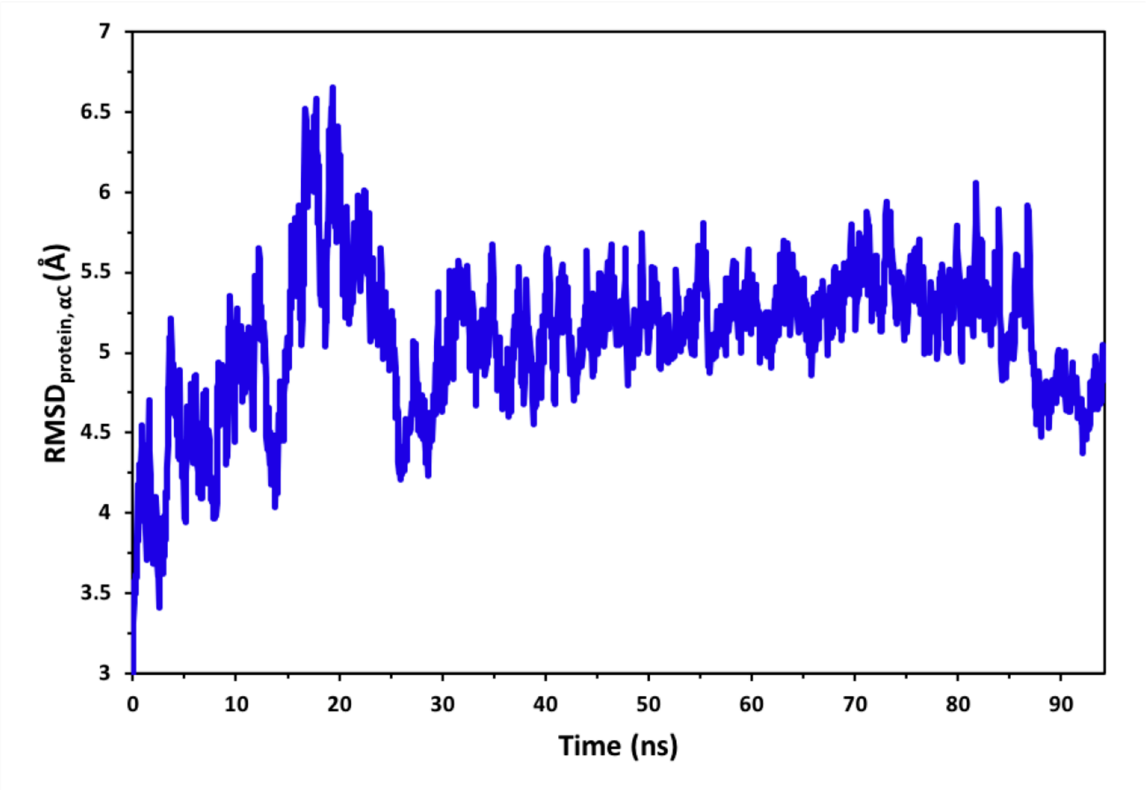

Fig. 4 ESI. RMSD of the membrane-free simulation.

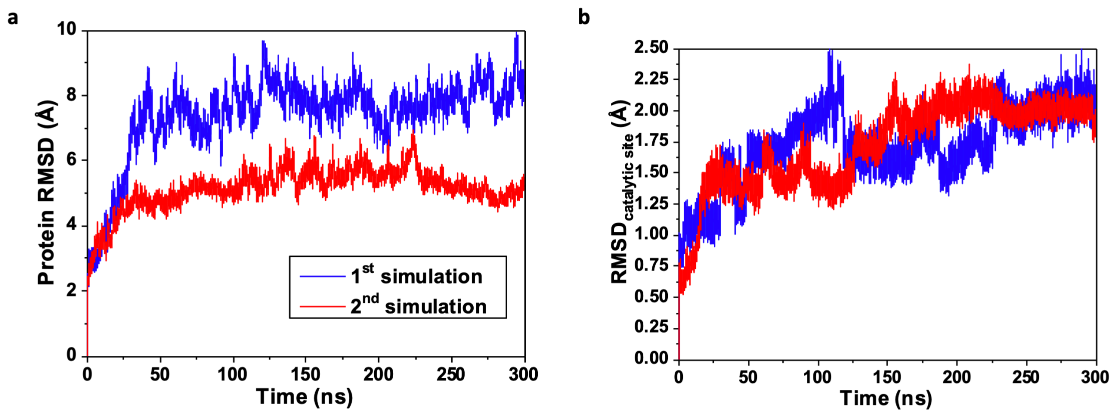

Fig. 5 ESI. RMSD of the membrane-embedded protein (**a**) in simulation 1 and simulation 2 and of the active site only (**b**).

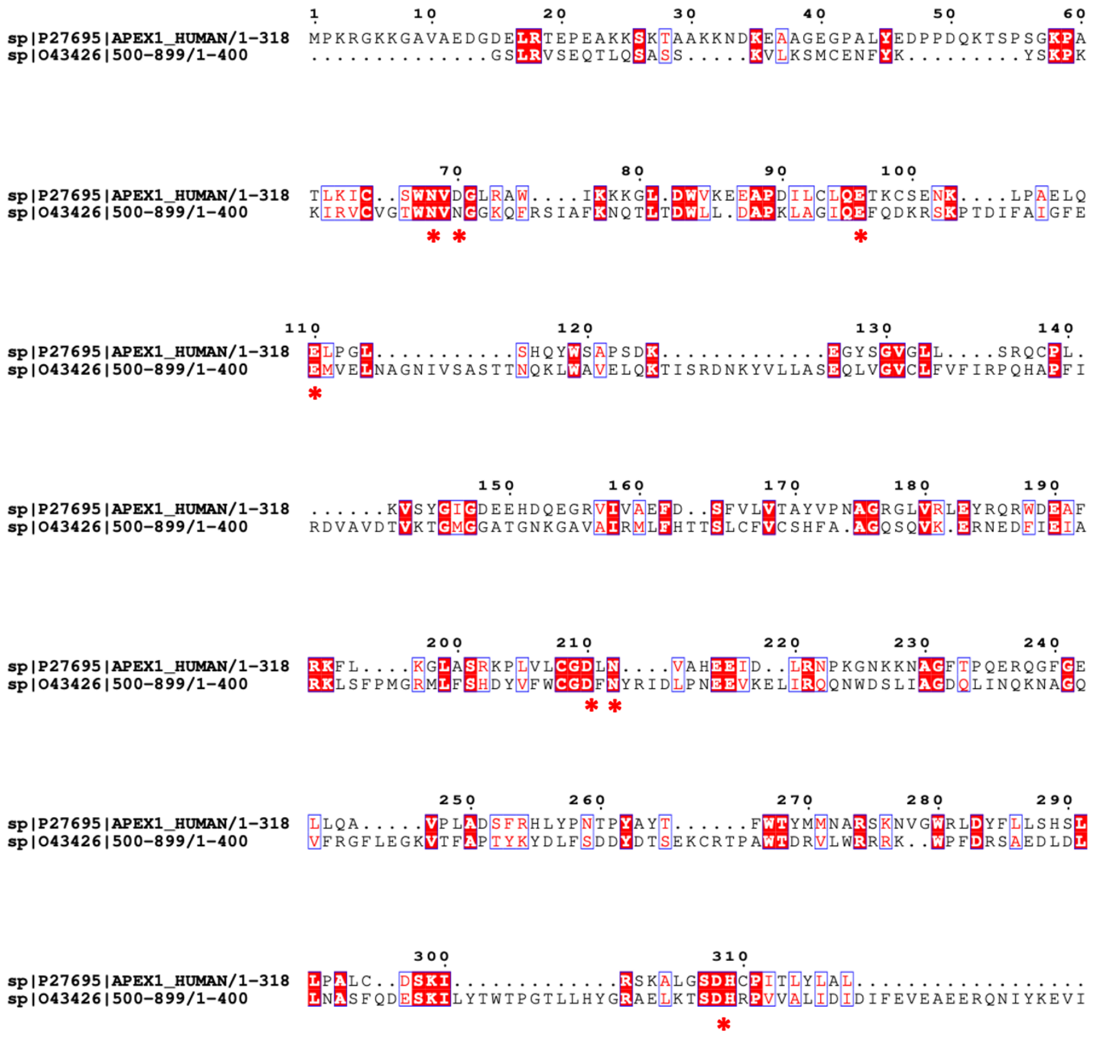

Fig. 6 ESI. Amino acid sequence of the 5-phosphatase domain of synj1 aligned to the apurinic/apyrimidinic base excision repair endonuclease Ape1. All sequences are obtained from Uniprot [13], and correspond to Homo sapiens. Visualised with Espript 3.0 [12].

References

[1] Balakrishnan R, Park J, Karra K, Hitz BC, Binkley G, Hong EL, et al. YeastMine-An integrated data warehouse for Saccharomyces cerevisiae data as a multipurpose tool-kit. Database 2012. https://doi.org/10.1093/database/bar062.

[2] Khurana V, Peng J, Chung CY, Auluck PK, Fanning S, Tardiff DF, et al. Genome-Scale Networks Link Neurodegenerative Disease Genes to ∝-Synuclein through Specific Molecular Pathways. Cell Syst 2017. https://doi.org/10.1016/j.cels.2016.12.011.

[3] Melnik, A., Cappellutti, V., Vaggi, F., Piazza, I., Tognetti, M., Soste, M., de Souza, N., Csikasz-Nagy, A., Piccotti P. In Preparation 2019.

[4] Kuhn M, von Mering C, Campillos M, Jensen LJ, Bork P. STITCH: Interaction networks of chemicals and proteins. Nucleic Acids Res 2008. https://doi.org/10.1093/nar/gkm795.

[5] Tsujishita Y, Guo S, Stolz LE, York JD, Hurley JH. Specificity determinants in phosphoinositide dephosphorylation: Crystal structure of an archetypal inositol polyphosphate 5-phosphatase. Cell 2001. https://doi.org/10.1016/S0092-8674(01)00326-9.

[6] Trésaugues L, Silvander C, Flodin S, Welin M, Nyman T, Gräslund S, et al. Structural basis for phosphoinositide substrate recognition, catalysis, and membrane interactions in human inositol polyphosphate 5-phosphatases. Structure 2014. https://doi.org/10.1016/j.str.2014.01.013.

[7] Hsu FS, Mao Y. The structure of phosphoinositide phosphatases: Insights into substrate specificity and catalysis. Biochim Biophys Acta - Mol Cell Biol Lipids 2015;1851:698–710. https://doi.org/10.1016/j.bbalip.2014.09.015.

[8] Mills SJ, Silvander C, Cozier G, Trésaugues L, Nordlund P, Potter BVL. Crystal Structures of Type-II Inositol Polyphosphate 5-Phosphatase INPP5B with Synthetic Inositol Polyphosphate Surrogates Reveal New Mechanistic Insights for the Inositol 5-Phosphatase Family. Biochemistry 2016. https://doi.org/10.1021/acs.biochem.5b00838.

[9] Clustalw U, To C, Multiple DO. ClustalW and ClustalX. Options 2003. https://doi.org/10.1002/0471250953.bi0203s00.

[10] Edgar RC. MUSCLE: Multiple sequence alignment with high accuracy and high throughput. Nucleic Acids Res 2004. https://doi.org/10.1093/nar/gkh340.

[11] Waterhouse AM, Procter JB, Martin DMA, Clamp M, Barton GJ. Jalview Version 2-A multiple sequence alignment editor and analysis workbench. Bioinformatics 2009. https://doi.org/10.1093/bioinformatics/btp033.

[12] Gouet P, Courcelle E, Stuart DI, Métoz F. ESPript: Analysis of multiple sequence alignments in PostScript. Bioinformatics 1999. https://doi.org/10.1093/bioinformatics/15.4.305.

[13] Hancock JM, Zvelebil MJ, Zvelebil MJ. UniProt. Dict. Bioinforma. Comput. Biol., 2004. https://doi.org/10.1002/9780471650126.dob0721.pub2.
